# Seed biostimulant *Bacillus* sp. MGW9 improves the salt tolerance of maize during seed germination

**DOI:** 10.1186/s13568-021-01237-1

**Published:** 2021-05-25

**Authors:** Heqin Li, Haiwang Yue, Li Li, Yu Liu, Haiyan Zhang, Jianhua Wang, Xuwen Jiang

**Affiliations:** 1 Dryland Technology Key Laboratory of Shandong Province, Qingdao Agricultural University, Qingdao, 266109 China; 2 Dryland Farming Institute, Hebei Academy of Agriculture and Forestry Sciences, Hengshui, 053000 China; 3 Seed Science and Technology Research Center, China Agricultural University, Beijing, 100193 China

**Keywords:** Salt stress, Seed biostimulant, Seed germination, *Zea mays*, *Bacillus* sp

## Abstract

Crop performance is seriously affected by high salt concentrations in soils. To develop improved seed pre-sowing treatment technologies, it is crucial to improve the salt tolerance of seed germination. Here, we isolated and identified the strain *Bacillus* sp. MGW9 and developed the seed biostimulant MGW9. The effects of seed biopriming with the seed biostimulant MGW9 in maize (*Zea mays* L.) under saline conditions were studied. The results show that the strain *Bacillus* sp. MGW9 has characteristics such as salt tolerance, nitrogen fixation, phosphorus dissolution, and indole-3-acetic acid production. Seed biopriming with the seed biostimulant MGW9 enhanced the performance of maize during seed germination under salinity stress, improving the germination energy, germination percentage, shoot/seedling length, primary root length, shoot/seedling fresh weight, shoot/seedling dry weight, root fresh weight and root dry weight. Seed biostimulant MGW9 biopriming also alleviated the salinity damage to maize by improving the relative water content, chlorophyll content, proline content, soluble sugar content, root activity, and activities of superoxide dismutase, catalase, peroxidase and ascorbate peroxidase, while decreasing the malondialdehyde content. In particular, the field seedling emergence of maize seeds in saline-alkali soil can be improved by biopriming with the seed biostimulant MGW9. Therefore, maize seed biopriming with the seed biostimulant MGW9 could be an effective approach to overcoming the inhibitory effects of salinity stress and promoting seed germination and seedling growth.

## Introduction

Soil salinization is an increasingly serious agricultural problem in the world. It greatly affects the growth and development of crops, resulting in a significant loss of productivity. As a result of poor irrigation, over-fertilization and desertification processes, cultivated soils around the world have become more saline and alkaline. More than 800 million hectares of land worldwide are currently affected by salt stress (Ramadoss et al. 2013). Maize (*Zea mays* L.) is an important global cereal crop whose production needs to be increased to meet the food needs of a growing world population (Tilman et al. 2011). Nevertheless, the growth of maize and grain quality can be severely affected by salinity, drought, high temperature and other adverse environmental conditions (Gong et al. 2014). Salt stress is emerging as a particular constraint to global crop production, and it is estimated that it will affect about 20% of the world's irrigated land and will lead to a loss of up to 50% of the land by the middle of the twenty-first century (Mahajan and Tuteja 2005; Zhu 2001). Sodium chloride (NaCl), as the main form of soil salinity, can lead to crop yield reduction or even death by making root water uptake more difficult, and can lead to plant poisoning by accumulating high concentrations of Na^+^ and Cl^−^ in plants (Deinlein et al. 2014; Paul and Lade 2014; Yu et al. 2020; Zhu 2001). High concentrations of salt lead to a combination of ionic imbalance and hypertonic effects at biochemical and molecular levels (Munns 2002; Tester and Davenport 2003). For example, salt stress leads to chloroplast damage, decreased photosynthetic rate and increased photorespiratory rate, accumulation of reactive oxygen species (ROS), decreased enzyme efficiency and activated *SOS* gene expression (Hoshida et al. 2000; Teixeira and Pereira 2007; Zhang et al. 2019). However, most plants have developed the ability to reduce the negative effects of salinity through the regulation and compartmentalization of ions, synthesis of compatible solutes, induction of antioxidant enzymes, induction of phytohormones, and alteration of photosynthetic pathways (Rojas-Tapias et al. 2012).

Various methodologies are in vogue for developing stress-tolerant varieties, either through conventional breeding or through transgenic technology. Alternatively, simpler and more economical practices are competing to solve this problem. Seed priming is a farmer-friendly technique recommended by many researchers for better establishment and growth even under adverse conditions (Filippou et al. 2013a; dos Santos Araújo 2021). It is well known that different environmental stresses often activate similar cell signaling pathways and cellular responses, and seed priming can activate these signaling pathways early in growth and lead to faster plant defense responses. Different seed priming methods employed to mitigate stress tolerance as reported by many researchers include: hydropriming, halo- and osmopriming, matrix priming, thermopriming, biopriming, drum priming, priming using growth regulators, nutrient priming and redox priming (Adhikary et al. 2021).

Some progress has been made in seed biopriming, and the growth-promoting ability of microorganisms may be highly specific to certain plant species, cultivars and genotypes (Bashan 1998; Moeinzadeh et al. 2010; dos Santos Araújo 2021). For example, *Trichoderma viride* and *T. harzianum* improved the seed germination and vigour of radish (Mukhopadhyay and Pan 2012). Seed biopriming with *T. viride* enhanced root growth in rice (Sureshrao et al. 2016). Seeds inoculated with *Bacillus subtilis* and *Pseudomonas fluorescens* produced a significantly increased in the fresh weight, dry weight, photosynthetic pigments, proline, total free amino acids and crude protein content in radish roots and leaves under salt stress (Mohamed and Gomaa 2012). Regarding seeds inoculated with *Hallobacillus sp.* SL3 and *Bacillus halodenitrificans* PU62, the root elongation and dry weight of wheat seedlings under salt stress were increased by more than 90 and 17.4%, respectively, compared with those of uninoculated wheat seedlings (Ramadoss et al. 2013). Under saline conditions, compared to uninoculated plants, inoculation with *Pseudomonas putida* KT2440 significantly improved seed germination and root and stem length of soybean and corn plants (Costa-Gutierrez et al. 2020). These findings suggest that seed biopriming with different beneficial microorganisms can not only improve seed quality, but also improve seedling vigour and resistance to abiotic stress, thus providing an innovative crop protection tool for the sustainable improvement of crop yields.

In recent years, plant biological stimulants have been developed which can help crops to resist abiotic stress, thus attracting much attention (Akhtar et al. 2008; Porcel et al. 2012; Woo and Pepe 2018). The widely accepted definition of a plant biostimulant is any substance and/or microorganism applied to plants with the aim of enhancing nutrition efficiency, abiotic stress tolerance and/or crop quality traits, regardless of its nutrient content (du Jardin 2015). In recent years, the use of biostimulants to improve crop seed germination and emergence ability under abiotic and biotic stress has attracted much attention (Selvakumar et al. 2017; Costa-Gutierrez et al. 2020; Li et al. 2020; Rafiq et al. 2020). Our purpose was to isolate and identify the salt-tolerant beneficial strains to develop seed biostimulants, and to study the effects of biopriming on the seed germination and seedling emergence of different maize varieties under salt stress by means of a pre-sowing treatment with the probiotics as bio-initiators, so as to provide a basis for the research on improving seed quality.

## Materials and methods

### Isolation and cultivation of strains

Bacterial strains were isolated from extremely arid soil samples near the Great Wall of the Ming Dynasty in Shandan County of Gansu Province (100.88E, 38.84 N). In August 2017, soil samples were put into sterile sealed bags, taken back to the laboratory and stored at − 20 ℃ for use. After being mixed and grounded, 20 g of the soil sample was poured into a triangular flask containing 80 mL of sterile water on a rotating shaker at 180 rpm for 30 min at room temperature. Then, the supernatant was diluted to 10^−6^ after a ten-fold dilution with deionized water. An aliquot of 200 μL of each dilution was spread on beef extract peptone agar medium containing 10 different concentrations of NaCl (5, 7, 8, 9, 10, 11, 12, 13, 14 and 15% (w/v)), with six replicates per concentration. After 72 h of culture at 28 ℃, the colonies with different morphological characteristics were selected and purified on the nutrient agar plate by the plate streaking method. The candidate bacterial strains were numbered into sterile tubes with 25% (v/v) glycerol and stored at -80 ℃.

### Screening for salt-tolerance level and growth promoting characteristics of strain MGW9

To a flask containing 50 mL of nutrient broth, NaCl was added to give a final salt concentration of 5, 7, 8, 9, 10, 11, 12, 13, 14 and 15% (w/v). The strains of active growth were then added to each flask and incubated on a rotating shaker at 30 ℃ and 180 rpm. Bacterial growth was determined as OD_600nm_ to determine salt tolerance again. In this study, the strain MGW9 and other candidate bacterial strains (data not shown) with a high salt-tolerance level were chosen for further study.

The strain MGW9 was streaked and inoculated into a nitrogen-free culture medium (Liu et al. 2017) containing 12% NaCl with three times, and was cultured in a dark incubator at 30 °C for 4–6 days. The nitrogen-fixing capacity was detected according to the presence or absence of colonies on a plate.

The strain MGW9 was incubated on bacterial inorganic and organic phosphorus media (Tao et al. 2008) containing 12% NaCl with three replicates at 30 °C for 7 days, respectively. Then, according to the presence of transparent circles, i.e., phosphate-solubilizing circles, if a clear area appeared around the colony, the property of dissolving phosphate was shown. The diameter of the phosphate-solubilizing zone (D) and colony diameter (d) were measured, and the phosphate-solubilizing ability of strain MGW9 was qualitatively tested by D/d. According to the method described by Li et al. (2019b), the phosphate solubilization index (PSI) = (colony diameter + halo zone diameter)/colony diameter.

According to the method of the indole-3-acetic acid (IAA) production test described by Li and Jiang (2017), King’s B medium containing 100 mg/mL L-tryptophan and 12% NaCl was used to screen for IAA production. The culture supernatant of the candidate strain was mixed with Salkowski reagent at a ratio of 1:1 (v:v). A pink mixture indicated the generation of IAA and its density was recorded at OD_530nm_. The concentration of IAA produced was estimated from a standard curve of IAA in the range of 0–100 μg/mL.

### Identification of strain MGW9

The strain MGW9 was cultured on beef extract peptone agar medium at 30 ℃ for 48 h at 200 rpm with three replicates, and then its morphological characteristics were observed by microscope. Genomic DNA of the strain MGW9 was extracted by bacterial genomic DNA rapid isolation kit (Sangon Biotech (Shanghai) Co., Ltd., China), and was identified according to the complete 16 s rDNA sequence using a forward 27F primer (5'-AGAGTTTGATCCTGGCTCAG-3') and the reverse 1492R primer (5'-GGTTACCTTGTTACGACTT-3') for PCR amplification reaction. The PCR products were sequenced by Qingdao Pacino Gene Biotechnology Co., Ltd. Sequence homology of nucleotides was compared using the blast search program. The tightly related sequences were aligned by ClustalW using the MEGA version 5.1 software package, and the phylogenetic tree was constructed by the neighbor joining (NJ) method. The bootstrap replications (1000) were used as statistical support for nodes in the phylogenetic tree.

### Seed priming using the seed biostimulant MGW9

Seeds of hybrid maize ‘Zhongdi175’ (ZD175), ‘Zhengdan958’ (ZD958) and ‘Denghai605’ (DH605) were used. Pure maize seeds were randomly selected from each sample for the following experiments.

(i) Thousand-seed weight (TSW) test: the TSW was measured using 500 seeds in each of the three replicates and then converted to thousand seed weight (Li et al. 2019a).
(ii) Seed moisture content (SMC) test: the seeds were ground and dried at 130 ± 0.5 ℃ for 4 h and the moisture content basis was calculated from the fresh weight (ISTA 2007).
(iii) Seed water absorption test: 100 maize seeds from each of the three maize varieties were measured for their initial weight, and then soaked in sterile water and taken out every 2 h. After the water on the surfaces of the seeds was wiped off, the seeds were weighed, and then the water absorption of the seeds at different time points was calculated, with three replicates for each variety. Additionally, the water absorption characteristic equation of maize seed was obtained by curve fitting analysis of the average water absorption of three maize seed samples at each time point.
(iv) Preparing SB-MGW9: the strain MGW9 was inoculated into a nutrient broth and cultured in a fermentation tank at a stirring speed of 150 rpm, a culture temperature of 28 ℃ and a ventilation rate of 1.5 L/min for 48–60 h, and the number of the MGW9 was adjusted to be 1.0 × 10^8^–1.5 × 10^8^ cfu/mL, while the pH value of the bacterial liquid was adjusted to be 7.0–8.0.
(v) Seed priming with SB-MGW9: two-factor randomized block design was used in the experiment. The soaking time, as factor A, was set to two different times: 3 and 6 h. The moisturizing time was factor B, which was divided into two different times: 12 and 24 h. After treatment, the primed seeds were air dried at 25 °C to near their original moisture contents. Factors A and B were randomly divided into 4 treatments, and the untreated group (no priming) was used as the control (C). Treatment 1 (T1) means that the seeds were soaked for 3 h, and moisturized for 12 h; Treatment 2 (T2) means that the seeds were soaked for 3 h, and moisturized for 24 h; Treatment 3 (T3) means that the seeds were soaked for 6 h, and moisturized for 12 h; and treatment 4 (T4) means that the seeds were soaked for 6 h, and moisturized for 24 h.

### Germination and seedling growth test

The pure seeds were randomly selected for the standard germination test. The seed surface was sterilized with 1% NaClO (w/v, Beijing Chemical Reagent Company, Beijing, China) for 10 min, then washed three times with distilled water and air dried for use. The seeds were germinated by adopting a rolling paper germination. Firstly, two pieces of germinating paper (Anchor Paper Co., St Paul, MN, USA) were stacked and moistened by 100 mM NaCl solution, and the redundant water on the paper was removed by a towel. Secondly, the primed seeds were alternately placed on a germination paper bed, with directions of the seed holes being consistent, the paper bed was rolled up and placed into a self-sealing bag, and the seed hole ends were vertically placed into a Versatile Environmental Test Chamber (MGC-350HP, Shanghai Yiheng Technology Instrument Co., Ltd., Shanghai, China) at 25 ± 0.5 ℃ and with an illumination cycle of 12 h of light and 12 h of darkness. Each treatment was repeated three times, with 100 seeds per repetition (Jiang et al. 2016). The no priming seeds were used as the control. The germination energy (GE) and germination percentage (GP) were measured on the 4th and 7th days after the experiment was established. GP is the normal seedling number on the 7th day after seed planting (Li et al. 2019a). While counting the GP, 10 seedlings with uniform size were randomly selected to measure six indices, including shoot/seedling length (SL), primary root length (PRL), shoot/seedling fresh weight (SFW), shoot/seedling dry weight (SDW), root fresh weight (RFW) and root dry weight (RDW). For SDW and RDW, the plant tissue (shoot/seedling or root) was dried at 105 ± 0.5 ℃ for 8 h.

### Assay for biochemical index

After passing through a 2 mm sieve, the sand was sterilized and placed in plastic pots (volume 150 mL), with 100 g of sterilized sand per pot. The content of deionized water in the sterilized sand was 10% (v/w). Salt treatment was carried out by supplementing deionized water with NaCl at a final concentration of 100 mM. After the primed seeds were placed in the sand bed, the pot was sealed with transparent preservative film. No primed seeds were used as the control. There were three replicates for each treatment and 15 seedlings per replicate. The relative water content (RWC) of the leaf samples was determined, expressed as a percentage, referring to the method of Ghahfarokhi et al. (2015). The chlorophyll (Chl) content was measured by a SPAD502 Plus meter. The level of lipid peroxidation was determined by the content of malondialdehyde (MDA), the content of proline was determined by extraction with 3% 5-sulfosalicylic acid at room temperature, and the content of soluble sugar was measured by the anthrone-sulfuric acid method (Zhu et al. 2010). The root activity was determined by the triphenyltetrazolium chloride (TTC) method (Li et al. 2015). Approximately 500 mg of a fresh leaf sample was homogenized in 10 mL of 0.05 M phosphate buffer (pH 7.8) solution and centrifuged at 10,000 × g for 10 min (Li et al. 2019b). The supernatant was then collected and stored at 4 ℃ for use. The activities of superoxide dismutase (SOD), catalase (CAT), peroxidase (POD) and ascorbate peroxidase (APX) were measured (Chen and Asada 1992; Ghahfarokhi et al. 2015; Zhu et al. 2010).

## Field seedling emergence test

For field seedling emergence (FSE), the samples were sown in saline-alkali land in the Binzhou (soil salt content was 0.61%, pH was 7.7), Dongying (soil salt content was 0.54%, pH was 7.6), and Weifang (soil salt content was 0.56%, pH was 7.5) experimental bases, Shandong, China, in 2020. In this study, the row spacing, spacing between rows, and length of rows were 0.06, 0.06, and 0.60 m, respectively. The seeds were sown using the single seed sowing method with 10 seeds per row and 10 rows per repetition (continuous row, 100 seeds) in three repetitions. The arrangement of seeds was designed using the method of partition comparison. In June, the field emergence test was completed. FSE was measured at the three-leaf stage of maize. FSE (%) = [FSE-Binzhou (%) + FSE-Dongying (%) + FSE-Weifang (%)]/3.

### Statistical analysis

Data were analyzed by means of a one-way analysis of variance (ANOVA) using the SAS statistical software package (SAS Institute, 1999), followed by the calculation of the lowest significant differences (LSD). The work was completed in the Seed Science and Engineering Laboratory of Qingdao Agricultural University from March to September 2020.

## Results

### Isolation, identification and characteristics of strain MGW9

According to the morphological characteristics, 19 strains of salt-tolerant bacteria were isolated from soil samples. Among all of the isolated strains, the strain MGW9 has the maximum salt-tolerance level of 12% NaCl; furthermore, this strain has growth promoting characteristics. The characteristics of the strain MGW9 cultured under the condition of 12% NaCl mainly include the following three aspects: (i) Nitrogen fixation: the strain MGW9 was streaked on nitrogen-free medium and cultured in a dark incubator at 30 ℃ for 4–6 days, and MGW9 colonies were found on the plate. (ii) Phosphorus dissolution: the transparent circle of MGW9 could be observed on the third day after inoculation in the organic phosphate and inorganic phosphate medium, and the size of the transparent circle tended to be stable until the seventh day. When the transparent zone was stable, the ratio of the diameter of dissolving phosphorus zone (D) to the diameter of bacterial colony (d) was 1.7 in organic phosphorus medium and 1.95 in inorganic phosphorus medium. iii) IAA production: the standard curve equation of IAA concentration and absorbance change was Y = 0.025X + 0.001 (R^2^ = 0.985, Y represents the absorbance, and X represents the IAA concentration). The IAA production of the strain MGW9 in King’s B medium was 19.24 mg/L (Table 1).

**Table 1:** The performance for nitrogen fixation, phosphorus dissolution and indole-3-acetic acid production of the strain MGW9

Strain	Characteristics	Results
MGW9	Nitrogen fixation (Yes or No)	Yes
	Organic phosphorus dissolution	D/d = 1.7
	Inorganic phosphorus dissolution	D/d = 1.95
	IAA production	19.24 mg/L

**Fig. 1:**
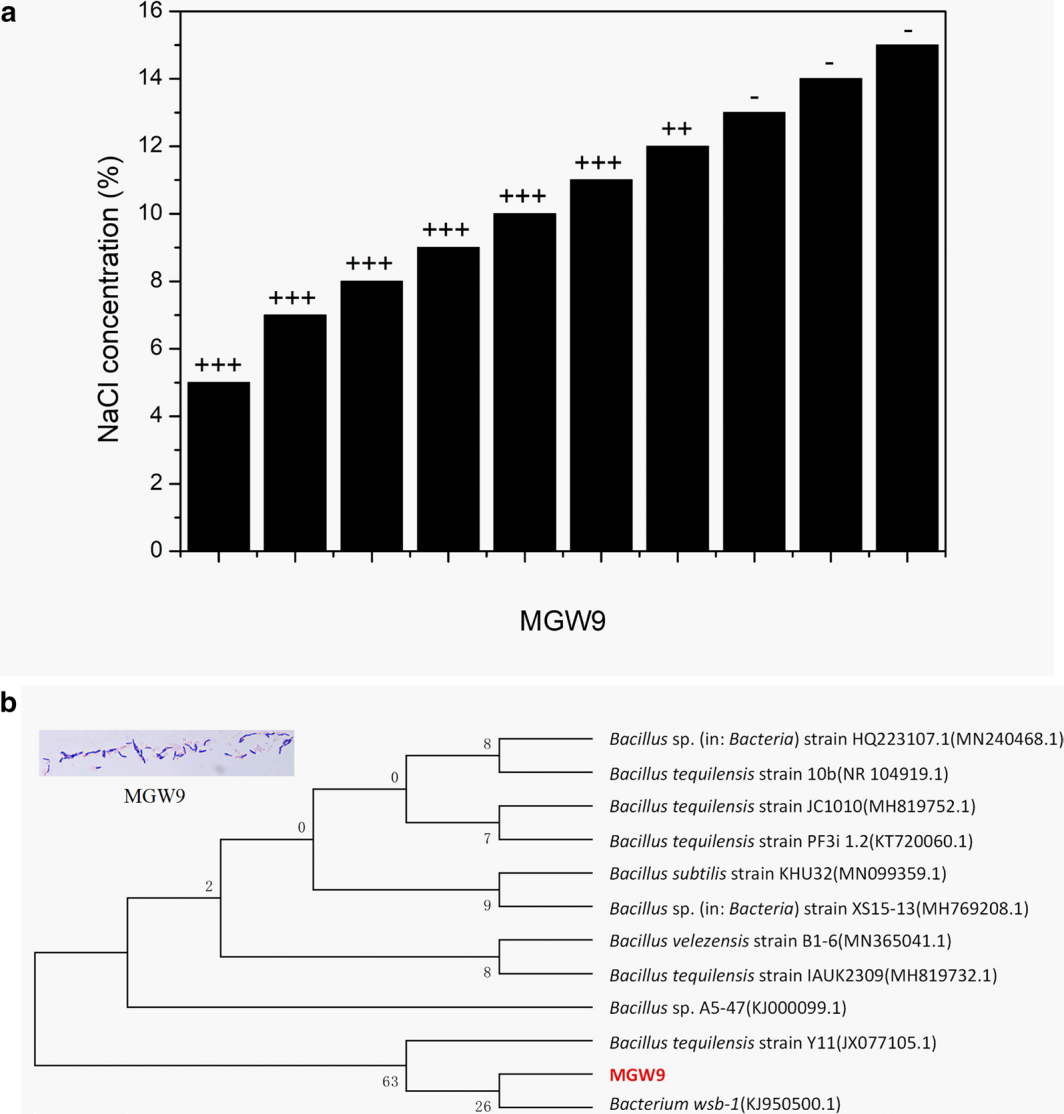
Identification and characteristics of the strain MGW9. **a** the salt-tolerance level of the strain MGW9 (From ‘ +  +  + ’ to ‘ + ’ indicates the salt tolerance ability from strong to weak; ‘-’ indicates no such ability.); **b** the morphology and phylogenetic tree of the strain MGW9

A BLAST search and phylogenetic analysis of the National Center for Biotechnology Information (NCBI) data showed that the strain MGW9 had 99.0% sequence homology with the bacteria *WSB-1* (KJ950500.1). Based on its morphology, including Gram-positive staining, the cells being rod-shaped and the 16S rDNA genetic sequence (1424 bp), the strain was named *Bacillus* sp*.* MGW9. Its 16S rRNA gene sequence has been deposited in the NCBI GenBank under accession number MW663489. The strain was preserved in the China General Microbiological Culture Collection Center on November 6, 2019; CGMCC No. 18690 (Fig. 1).

### The thousand seed weight, seed moisture content and water absorption characteristics of seed samples from three maize varieties

**Fig. 2:**
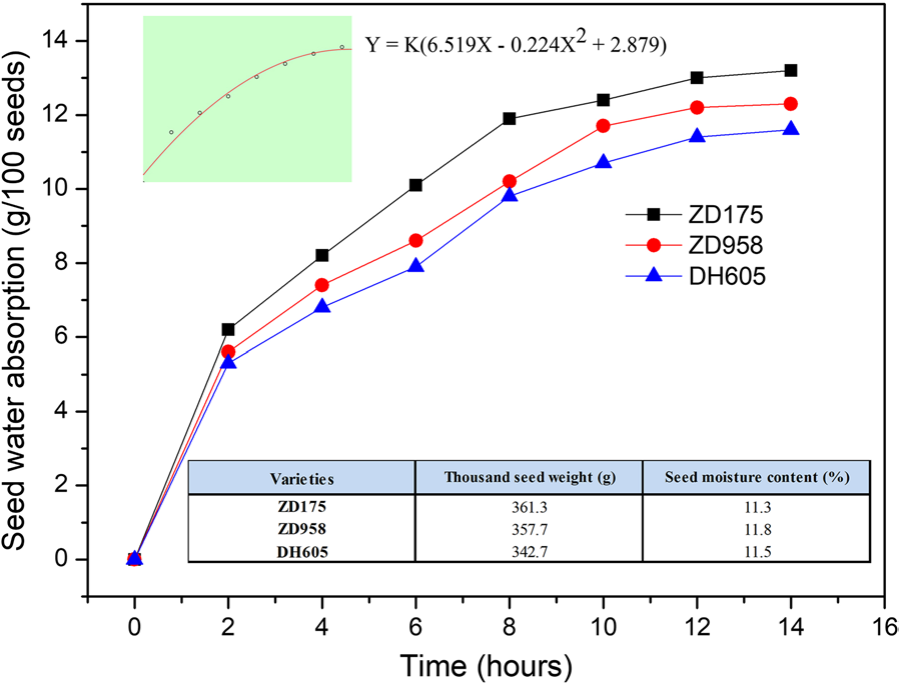
The initial indices and water absorption characteristics of maize seed samples

The thousand seed weight (TSW) and seed moisture content (SMC) of seed samples from three maize varieties were 342.7–361.3 g and 11.3–11.8% (lower than the safe water content 13%), respectively. The water absorption curve equation of maize seed was Y = K(6.519X − 0.224X^2^ + 2.879), where K is the coefficient of variation. In the seed imbibition stage, the water absorption rate of the seed samples of three varieties showed a trend of first fast and then slow change, and the change became stable after 12 h (Fig. 2).

### Effects of seed biopriming with biostimulant MGW9 on maize seed germination under normal and saline conditions

**Fig. 3:**
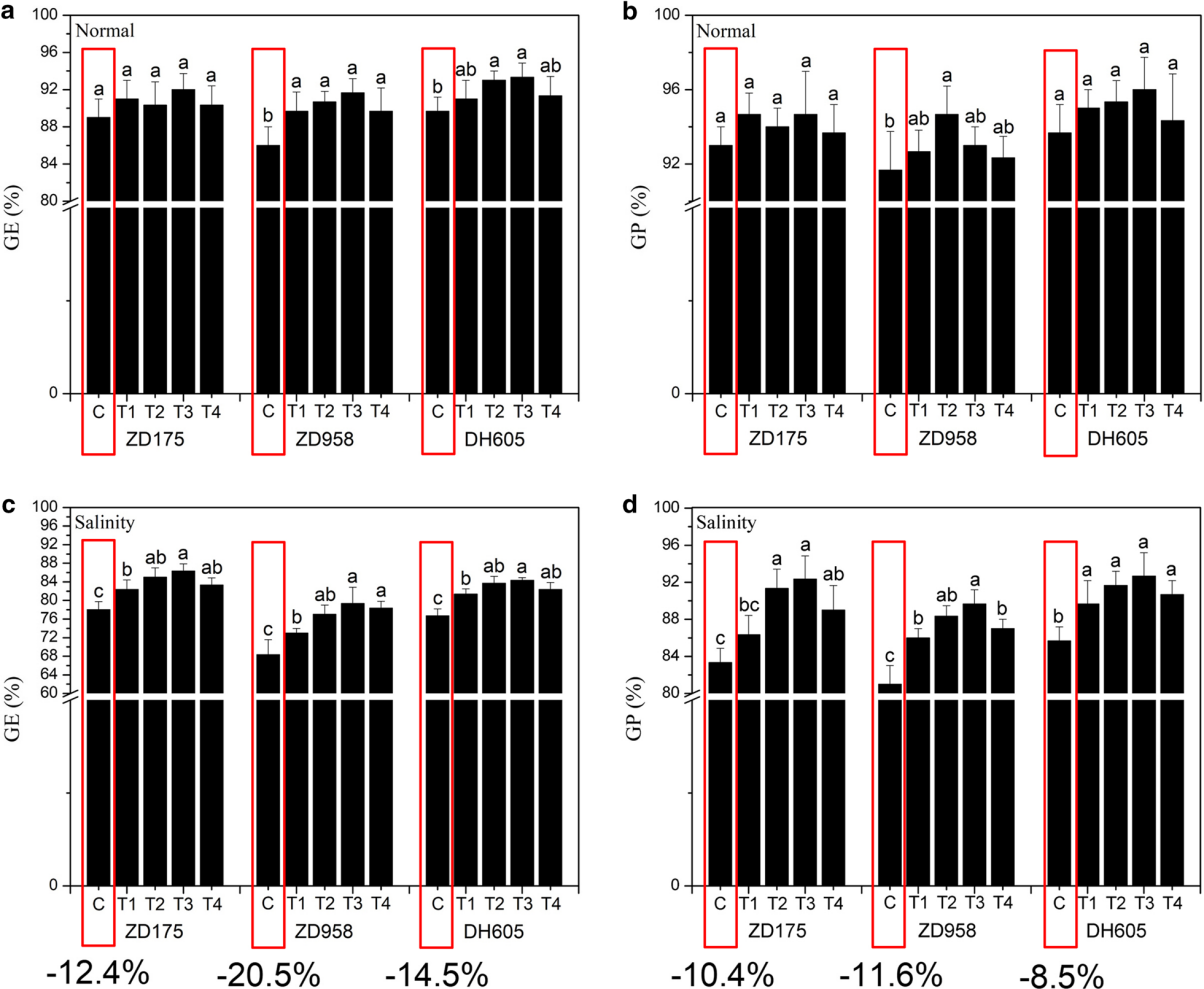
Effects of the seed biopriming with SB-MGW9 on maize seed germination under normal and saline conditions. The values represent the average of the data obtained in the experiment (n = 3). There was significant difference between treatments with different letters (*P* < 0.05). *GE* germination energy, *GP* germination percentage

Compared to normal conditions, the germination energy (GE) and germination percentage (GP) of three maize varieties sample seeds were significantly decreased under salt stress, the GE of ZD175, ZD958 and DH605 decreased by 5, 20.5 and 14.5%, respectively, and the GP of ZD175, ZD958 and DH605 decreased by 10, 11.6 and 8.5%, respectively. Compared with the control, the GE and GP of seeds after priming treatments were higher, and the priming effects were different under different germination environments. In the normal germination environment, except for ZD958, the GE and GP of ZD175 and DH605 sample seeds after biopriming were not significantly different from the control (*P* < 0.05). Additionally, there was no significant difference in the GE among different priming treatments of the same variety, and the GP was the same (Fig. 3a, b). This may be related to the fact that the priming effect was not obvious when the initial level of seed vigour of the sample was high. Under salinity stress, the GE and GP of seeds after priming treatments were significantly higher than that of the control (*P* < 0.05), except for the GP of ZD175-T1, and the priming effects of different priming treatments were different (Fig. 3c, d). Comprehensively analyzing the GE and GP of different priming treatments, the results show that T3 had the best seed biopriming effect (Fig. 3).

### Effects of seed biopriming with biostimulant MGW9 on maize seedling growth under salinity stress condition

Under salinity stress, the six seedling growth indices after seed biopriming were higher than the control. The suitable seed biopriming treatment with SB-MGW9 was different from different varieties. The suitable treatments of ZD175 were T2 and T3, those of ZD958 were T3 and T4, and those of DH605 were T2 and T3 (Table 2). According to comprehensive consideration of the six indices of seedling growth, the most suitable treatment for the three maize varieties was T3. Compared with the control, the shoot/seedling length (SL), primary root length (PRL), shoot/seedling fresh weight (SFW), root fresh weight (RFW), shoot dry weight (SDW), root dry weight (RDW) of ZD175 increased by 49.3, 50.7, 58.0, 70.0, 61.1 and 61.5%, respectively, ZD958 increased by 49.2, 49.2, 35.0, 25.0, 57.3 and 83.3%, respectively, and DH605 increased by 44.1, 46.5, 60.0, 62.5, 48.9 and 61.5%, respectively (Table 2).

**Table 2:** Effects of the seed biopriming with SB-MGW9 on the shoot/seedling length, primary root length, shoot/seedling fresh weight, shoot dry weight, root fresh weight and root dry weight in maize under salinity stress

Varieties	Treatments	SL	PRL	SFW	SDW	RFW	RDW
		(cm)	(cm)	(g/10S)	(g/10S)	(g/10S)	(g/10S)
ZD175	C	2.21 ± 0.02d	5.06 ± 0.13c	1.00 ± 0.01c	0.10 ± 0.02b	0.95 ± 0.06c	0.13 ± 0.01c
	T1	2.88 ± 0.10b	6.90 ± 0.62b	1.37 ± 0.07b	0.13 ± 0.02ab	1.24 ± 0.13b	0.17 ± 0.01b
	T2	3.05 ± 0.08b	7.85 ± 0.60a	1.46 ± 0.16ab	0.16 ± 0.03a	1.40 ± 0.07ab	0.21 ± 0.02a
	T3	3.30 ± 0.14a	8.08 ± 0.53a	1.58 ± 0.12a	0.17 ± 0.04a	1.53 ± 0.12a	0.21 ± 0.02a
	T4	2.67 ± 0.11c	7.48 ± 0.51ab	1.28 ± 0.14b	0.15 ± 0.01a	1.32 ± 0.12b	0.20 ± 0.01a
ZD958	C	1.93 ± 0.14c	5.11 ± 0.10d	0.80 ± 0.08d	0.08 ± 0.01c	0.82 ± 0.12c	0.12 ± 0.01d
	T1	2.38 ± 0.19ab	6.65 ± 0.36c	1.01 ± 0.03c	0.09 ± 0.02bc	1.13 ± 0.06ab	0.16 ± 0.02c
	T2	2.21 ± 0.07bc	7.22 ± 0.17b	1.15 ± 0.04b	0.11 ± 0.01ab	1.07 ± 0.05b	0.18 ± 0.02bc
	T3	2.73 ± 0.34a	7.73 ± 0.05a	1.08 ± 0.06bc	0.10 ± 0.002ab	1.29 ± 0.18a	0.22 ± 0.01a
	T4	2.52 ± 0.25ab	7.58 ± 0.51ab	1.29 ± 0.04a	0.12 ± 0.004a	1.22 ± 0.07ab	0.19 ± 0.02ab
DH605	C	1.98 ± 0.16c	4.54 ± 0.15d	0.90 ± 0.03d	0.08 ± 0.01c	0.92 ± 0.07b	0.13 ± 0.01d
	T1	2.27 ± 0.31bc	5.85 ± 0.19c	1.14 ± 0.04c	0.10 ± 0.03bc	1.26 ± 0.14a	0.18 ± 0.02c
	T2	2.59 ± 0.23ab	6.90 ± 0.22a	1.30 ± 0.10b	0.12 ± 0.002ab	1.45 ± 0.24a	0.23 ± 0.01a
	T3	2.68 ± 0.17a	6.65 ± 0.32b	1.44 ± 0.14a	0.13 ± 0.01a	1.37 ± 0.28a	0.21 ± 0.02ab
	T4	2.45 ± 0.10ab	6.34 ± 0.23b	1.22 ± 0.01bc	0.12 ± 0.004ab	1.21 ± 0.05ab	0.20 ± 0.01bc

According to Duncan's multiple range test, different letters in the same column indicate significant differences between treatments at the 0.05 level

*SL* shoot/seedling length, *PRL* primary root length, *SFW* shoot/seedling fresh weight, *RFW* root fresh weight, *SDW* shoot dry weight, *RDW* root dry weight, *S* seedling (s)

### Effects of seed biopriming with biostimulant MGW9 on the relative water content, chlorophyll content, malondialdehyde content, proline content, soluble sugar content and root activity of maize seedlings under salinity stress

**Fig. 4:**
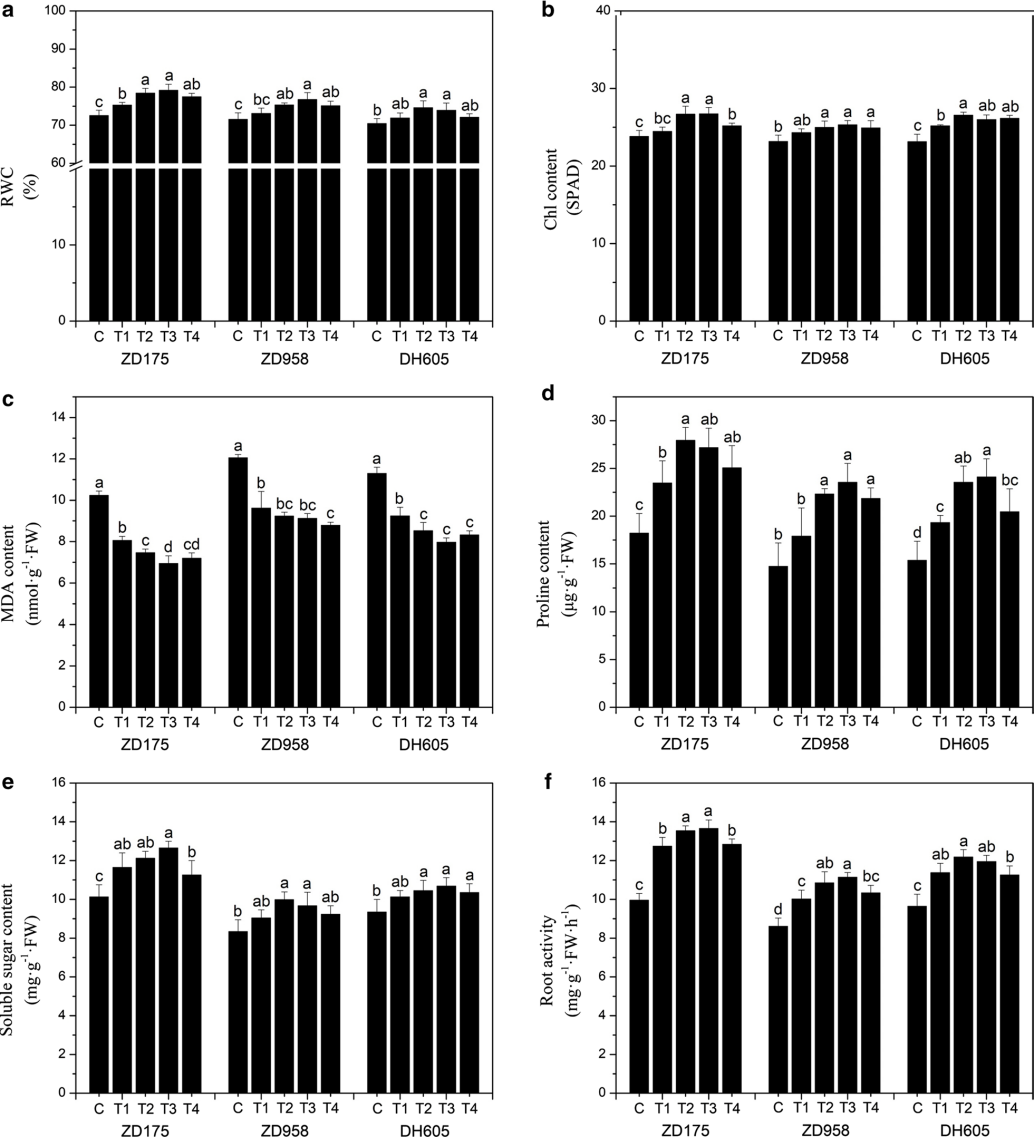
Effects of the seed biopriming with SB-MGW9 on the relative water content, chlorophyll content, malondialdehyde content, proline content, soluble sugar content and root activity in maize under saline conditions. The values represent the average of the data obtained in the experiment (n = 3). There was significant difference between treatments with different letters (*P* < 0.05). *RWC* relative water content, *Chl* chlorophyll, *MDA* malondialdehyde

Under salinity stress, compared with the control, the relative water content (RWC), chlorophyll (Chl) content, proline content, soluble sugar content and root activity of the seedlings of the three maize varieties after seed biopriming were significantly increased except for the malondialdehyde (MDA) content (*P* < 0.05). After comparative analysis of the data of the six biochemical indices, it can be seen that the different seed biopriming treatments have different effects. According to the content of MDA, the suitable seed biopriming treatments were T3 and T4, but there was no significant difference between T3 and T4. Additionally, regarding the other five indices, the suitable seed biopriming treatments for the three maize varieties were T2 and T3, and there was also no significant difference between T2 and T3. According to comprehensive consideration of the six biochemical indices data, the suitable seed biopriming treatment for the three maize varieties was T3. Compared with the control, the RWC, Chl, proline, soluble sugar content and root activity of ZD175-T3 increased by 9.1, 12.3, 49.1, 37.2 and 25.0%, respectively, and those of ZD958-T3 increased by 7.3, 9.3, 59.9, 29.4 and 15.9%, respectively, and those of DH605-T3 increased by 5.0, 12.3, 56.9, 24.0 and 14.3%, respectively. In addition, the MDA content in seedlings of ZD175-T3, ZD958-T3 and DH605-T3 decreased by 32.2, 24.3 and 29.4%, respectively (Fig. 4).

### Effects of seed biopriming with biostimulant MGW9 on the superoxide dismutase, catalase, peroxidase and ascorbate peroxidase activities of maize seedlings under salinity stress

**Fig. 5:**
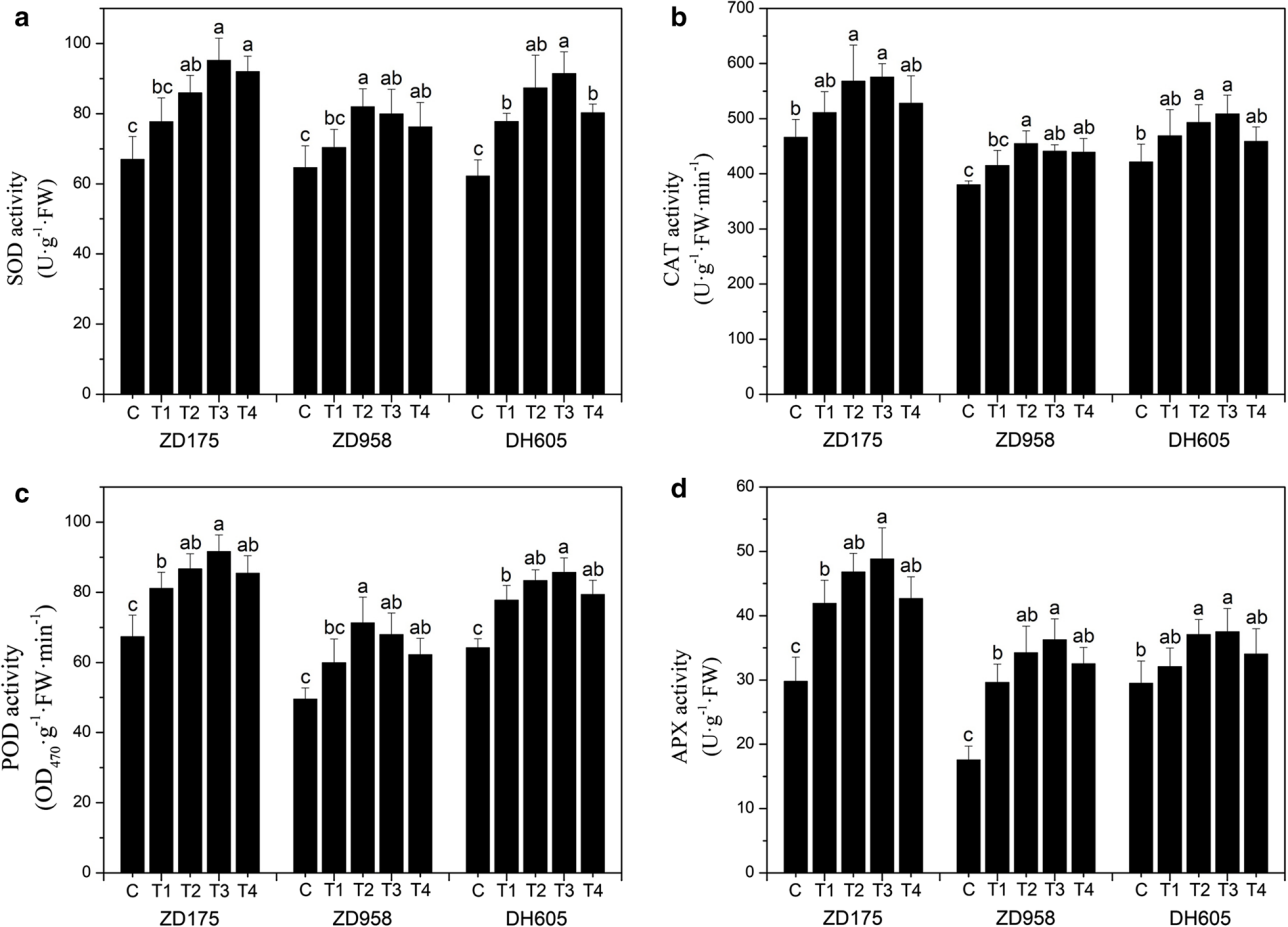
Effects of the seed biopriming with SB-MGW9 on the superoxide dismutase, catalase, peroxidase and ascorbate peroxidase activities in maize under saline conditions. The values represent the average of the data obtained in the experiment (n = 3). There was significant difference between treatments with different letters (*P* < 0.05). *SOD* superoxide dismutase, *CAT* catalase, *POD* peroxidase, *APX* ascorbate peroxidase, *U* active unit, *FW* fresh weight

Compared with the control, the activities of superoxide dismutase (SOD), catalase (CAT), peroxidase (POD) and ascorbate peroxidase (APX) in maize seedlings increased significantly after seed biopriming with SB-MGW9 (*P* < 0.05). According to comprehensive analysis of the four sets of enzyme activity data, the different seed biopriming treatments had different priming effects. The suitable seed biopriming treatments for the three maize varieties were T2 and T3, and there were no significant differences in the four enzyme activity indices between T2 and T3. The most suitable treatments for the different maize varieties were T3 for ZD175 and DH605, and T2 for ZD958. Compared with the control, the SOD, CAT, POD and APX activities of ZD175-T3 were increased by 42.1, 23.4, 36.1 and 63.9%, respectively; the SOD, CAT, POD and APX activities of ZD958-T2 were increased by 26.8, 19.6, 43.9 and 94.9%, respectively; and the SOD, CAT, POD and APX activities of DH605-T3 were increased by 47.0, 20.7, 33.5 and 27.2%, respectively (Fig. 5).

### Effects of seed biopriming with biostimulant MGW9 on the saline-alkali field seedling emergence of maize

**Fig. 6:**
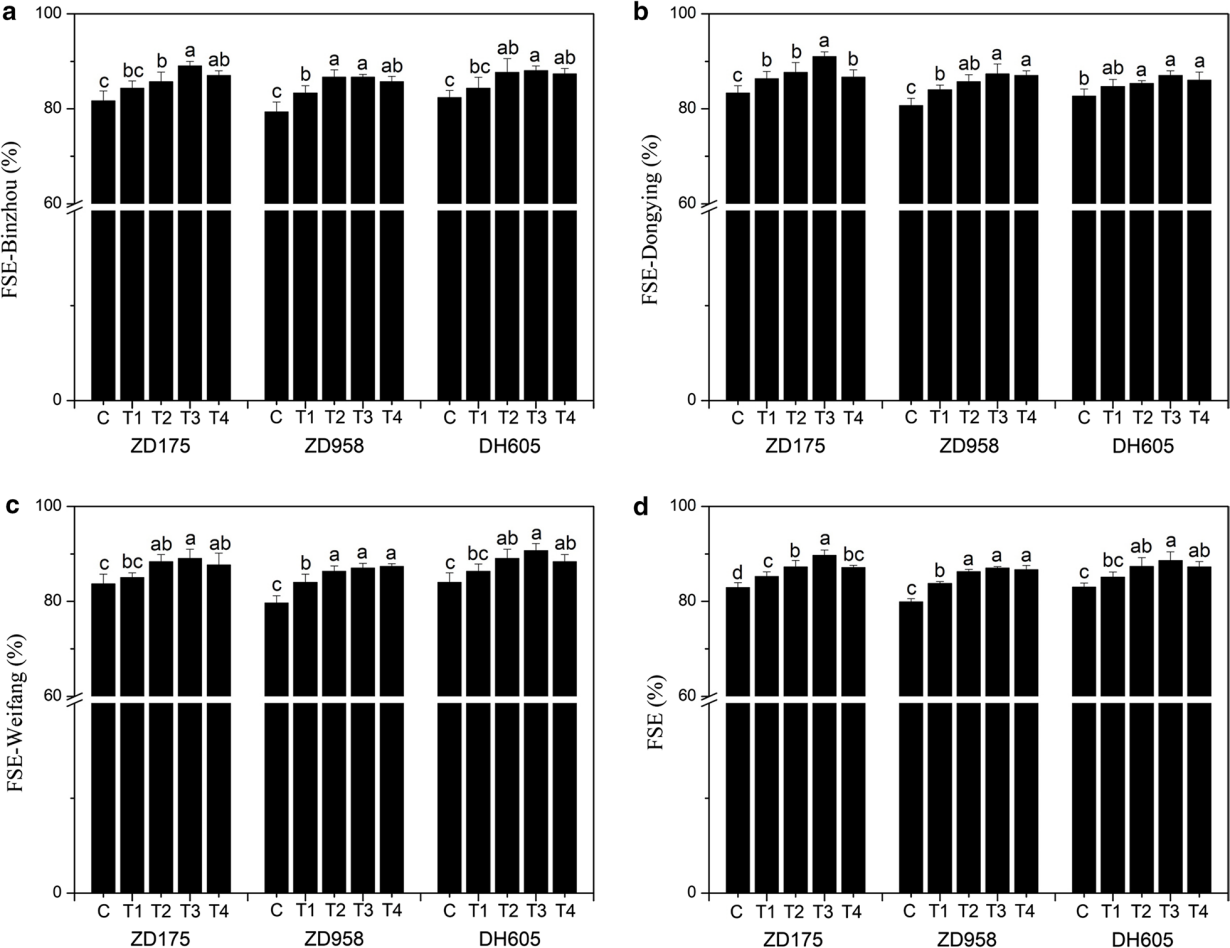
Effects of the seed biopriming with SB-MGW9 on the saline-alkali field seedling emergence of maize. The values represent the average of the data obtained in the experiment (n = 3). There was significant difference between treatments with different letters (*P* < 0.05). *FSE* field seedling emergence, *FSE (%)* [FSE-Binzhou (%) + FSE-Dongying (%) + FSE-Weifang (%)]/3

The results show that the field seedling emergence (FSE) of maize seeds after biopriming treatment increased significantly compared with the control (*P* < 0.05), and different maize varieties and biopriming treatments had different effects. According to the results of Binzhou-FSE, the suitable seed biopriming treatments for ZD175 were T3 and T4, while those for ZD958 and DH605 were T2 and T3; The suitable seed biopriming treatments were T2 and T3 for ZD175, and T3 and T4 for ZD958 and DH605 according to the results of Dongying-FSE; and the results of Weifang-FSE showed that the suitable seed biopriming treatments of ZD175 and DH605 were T2 and T3, while those for ZD958 were T3 and T4. According to the results of the FSE, which is the average of FSE-Binzhou, FSE-Dongying and FSE-Weifang, the suitable seed biopriming treatments for ZD175 and ZD958 were T3 and T4, and for DH605 they were T2 and T3. Comprehensively considering the results of these indices, the most suitable seed biopriming treatment for the three maize varieties was T3. Compared to the control, FSE-ZD175-T3, FSE-ZD958-T3 and FSE-DH605-T3 increased by 8.2, 8.9 and 6.7%, respectively (Fig. 6).

## Discussion

Soil salinity is an increasingly serious global problem, as salt hampers plant growth and development and reduces crop yield. Seed germination and early seedling growth are critical stages in plant establishment and production and are very sensitive to salt stress. The harmful effects of NaCl on seed germination and seedling emergence are caused by the decrease in water use efficiency and nutrient supplement ability when sodium accumulates in soil and the toxic effects of sodium and chloride ions on plants occur (Parida and Das 2005; Munns and Tester 2008; Deinlein et al. 2014; van Zelm et al. 2020). This study indicated that 100 mM NaCl solution had an obvious salt stress effect on the seed germination and seedling growth of three maize varieties, ZD175, ZD958 and DH605. The germination energy (GE) and germination percentage (GP) of the seeds under salt stress conditions are, respectively, reduced by 12.4–20.5 and 8.5–11.6% compared with the control (no stress). Therefore, it is of great practical significance for agricultural production to study the technical methods of improving seed vigour under salt stress in order to alleviate the adverse effects of salt stress on seed germination and seedling emergence.

Various methods have been used to improve crop resistance to stress, including conventional breeding methods such as selective hybridization, mutation breeding, polyploid breeding, genetic engineering etc. (Jisha and Puthur 2016), but seed priming as a simple, economical and effective method is more popular with farmers, as it can stimulate seed germination, enhance morphological parameters, and improve plant growth and development under abiotic stress (Jisha et al. 2013; Rhaman et al. 2020a, 2020b). For instance, dos Santos Araújo et al. (2021) suggested that seed priming with H_2_O_2_ can improve the salt tolerance of maize plants by protecting the chloroplast ultrastructure and regulating primary metabolites. In addition, seed priming has been reported to improve the salt tolerance of maize (Li and Jiang 2017), wheat (Ali et al. 2017), cucumber (Maach et al. 2021), camelina (Huang et al. 2021) and other crops.

A recent trend in sustainable development is the use of beneficial microorganisms to increase the nutrient use efficiency of field crops without compromising soil health (Meena et al. 2017). Biopriming is an emerging and promising seed and/or seedling treatment tool for inducing systemic resistance to abiotic and biotic stresses in treated crop. It is a process of biological treatment of seeds that involves combining seed hydration and inoculation with beneficial organisms to protect seeds (Rakshit et al. 2015). In most cases, microbial inoculants such as rhizospheric or endophytic microorganisms (bacteria or fungi) that promote plant growth are used (Ogireddy et al. 2019; Rakshit et al. 2015). As with other seed priming techniques, this technique has proven to be of paramount importance in improving seed quality and performance, as well as plant growth (Aliye et al. 2008; Rajkumar et al. 2010, 2012).

Plant biological stimulants are a new concept put forward in recent years. They are applied to plants for the purpose of enhancing nutritional efficiency, abiotic stress tolerance and/or crop quality traits, irrespective of their nutritional content. By definition (du Jardin 2015), beneficial microorganisms are one of the most important sources for the development of plant biostimulant products. Regarding beneficial fungi, some have been extensively studied and used for their biopesticidal and biocontrol (inducer of disease resistance) abilities and have been exploited by the biotechnology industry as sources of enzymes (Mukherjee et al. 2012; Nicolás et al. 2014). Many plant responses have been demonstrated to be fungus induced, including abiotic stress tolerance, high nutrient use efficiency and high plant growth performance (Colla et al. 2015; Shoresh et al. 2010). Based on the effects, these fungi can be considered as biostimulants. Regarding beneficial bacteria, they can interact with plants in all possible ways. There are two main types of symbiotic endosymbionts and symbiotic rhizospheric plant growth-promoting rhizobacteria (PGPRs) when they are used as biostimulants. PGPRs are multifunctional, affecting all aspects of plant life, including nutrition and growth, morphogenesis and development, responses to biotic and abiotic stresses, and interactions with other organisms in agroecosystems (Babalola 2010; Berendsen et al. 2012; Berg et al. 2014; Bhattacharyya and Jha 2012; Philippot et al. 2013). Some of these functions are usually performed by the same organism, some are strain-specific, and others depend on synergy in the bacterial community. At present, the useful of plant biostimulants to improve seed germination and seed vigour has become a research hotspot. Therefore, in this study, *Bacillus* sp*.* MGW9 was isolated and purified from extremely arid soil samples by salt-tolerant screening and morphological and molecular identification. Based on the characteristics of salt tolerance, nitrogen fixation, phosphorus solubilization and indole-3-acetic acid (IAA) production of *Bacillus* sp*.* MGW9, the strain was used to develop the seed biostimulant MGW9 (SB-MGW9).

The objective of this study was to investigate the effects of SB-MGW9 biopriming on the seed germination and seedling growth of maize under salt stress. Related reports have shown that some microorganisms could improve the growth performance of plants under a stress environment by providing plant hormones, soluble phosphate, fixed nitrogen, and other substances (Hayat et al. 2010; Ji et al. 2014), and the characteristics of the strains are similar to *Bacillus* sp*.* MGW9. Some researchers began to pay attention to the application of microorganisms in seed pre-sowing treatment because of the ability of beneficial microorganisms to inhibit diseases and improve crop germination ability and vitality. We set up four seed biopriming treatments according to the water absorption characteristics of maize seeds (Fig. 2), including seed soaking time and moisturizing time. The results of the germination test show that the germination energy (GE) and germination percentage (GP) of three maize varieties under normal and salt stress conditions were increased after seed biopriming treatment (Fig. 3), but the GE and GP of the seed biopriming treatments of ZD175 and the GP of seed biopriming treatments of DH605 were not significantly different from the control under normal conditions (Fig. 3a). However, under saline condition, the GE and GP of T2, T3 and T4 of ZD175 and those of the four seed biopriming treatments of ZD958 and DH605 were significantly higher than that of the control (*P* < 0.05) (Fig. 3b). Our results show that SB-MGW9 biopriming could improve the GE and GP of maize seeds under NaCl stress, and if the initial level of seed vigour is high and the seeds germinate under normal conditions, the effects of SB-MGW9 biopriming may not be significant. This may be due to the earlier completion of metabolic activities before germination in the priming process (dos Santos Araújo et al. 2021; Panuccio et al. 2018), and this advantage of priming seeds leads to the improvement of seed germination and seedling growth.

Salt stress often induces the increase in reactive oxygen species (ROS), hydrogen peroxide (H_2_O_2_), superoxide anions (O^2−^) and hydroxyl radicals (·OH) in plants, resulting in oxidative damage to plants (Hyodo et al., 2017). Malondialdehyde (MDA) is a kind of lipid peroxidation product, which is considered to be one of the important indices of oxidative damage to cell membrane caused by ROS (Parida and Das 2005). Our results show that salt stress induced an increase in MDA content in maize seedlings, suggesting that the presence of salt stress may enhance membrane lipid peroxidation, leading to increased membrane permeability, electrolyte extravasation, and ultimately damage to the cell membrane system. However, ZD175-T3, ZD958-T4 and DH605-T3 significantly decreased by 32.2, 27.1 and 29.4%, respectively, compared to non-bioprimed seeds (Fig. 4c). This indicated that maize seedlings had stronger tolerance to oxidative stress after seed biopriming with SB-MGW9. This is similar to the result showing that the content of MDA in mycorrhizal inoculated maize plants is lower than that in non-mycorrhizal plants under temperature stress (Zhu et al. 2010). In addition, proline is a good osmotic agent and radical scavenger to stabilize subcellular structure, which can quench single O^2−^ or directly react with OH (Filippou et al. 2013b). Under stress conditions, proline accumulation may be due to increased synthesis and decreased degradation, which helps to maintain cell water status and protect cell membranes and proteins (Kishor and Sreenivasulu 2014). Bano and Fatima (2009) showed that microorganisms (*Rhizobium* and *Pseudomonas*) introduced in the rhizosphere can improve the water use efficiency of maize plants, induce the synthesis of osmotic regulators such as proline, and help maintain the integrity of cell membranes. This is consistent with the results of this study under salt stress, the proline content of biopriming maize seedlings was significantly higher than that of non-biopriming seedlings (Fig. 4d).

In plants, carbohydrate metabolism is involved in key processes in response to abiotic stresses, with key roles in carbon storage, osmotic homeostasis, osmoprotectants and free radical scavenging (Gangola and Ramadoss 2018). Soluble sugars are important osmolytes in plant cells, and their accumulation contributes to the regulation of osmotic stress in plant cells and results in the preservation of biomolecules and membranes (Bohnert and Sheveleva 1998). Gandonou et al. (2012) reported a significant increase in soluble sugar content in sugarcane leaves and roots under salt stress. Borrelli et al. (2018) showed that carbohydrate stores in wheat plants under salt stress are quickly mobilized, releasing soluble sugars that act as compatible solutes under stress. In this work, our results show that the soluble sugar content increased significantly after seed biopriming with SB-MGW9, which could help to alleviate osmotic stress (Fig. 4e). Similar results were observed by Feng et al. (2002), who found that the colonization of arbuscular mycorrhizal fungi could significantly increase the soluble sugar content of salt-treated maize seedlings, indicating that these plants have a higher osmotic adjustment capacity. Additionally, dos Santos Araújo et al. (2021) showed that H_2_O_2_ priming could increase the contents of six sugars and polyols in maize plants under salt stress to alleviate osmotic stress.

In addition, plants have evolved a series of defensive measures, including the use of their own antioxidant enzymes and non-antioxidant metabolites to eliminate ROS (Feng et al. 2002). The common antioxidant enzymes include SOD, POD, CAT and APX, which play an important role in the process of scavenging ROS. SOD catalyzes the conversion of O^2−^ to H_2_O_2_ and O_2_, while POD and CAT can scavenge H_2_O. Additionally, APX can protect chloroplasts and other cellular components from H_2_O_2_ and hydroxyl damage. Some studies have found that under abiotic stress, inoculation of some beneficial bacteria (such as *Bacillus subtilis* SU47, *Glomus etunicatum*) can improve plant antioxidant enzyme activity to alleviate the negative effects of stress on plant growth (Upadhyay et al. 2011; Zhu et al. 2010). In our study, compared with non-bioprimed seeds, the activities of SOD, POD, CAT and APX in maize seedlings were significantly increased after biopriming treatment (*P* < 0.05), indicating that SB-MGW9 biopriming could improve the antioxidant defense capacity of maize seedlings under salt stress (Fig. 5).

In summary, based on the results of the RWC, the content of chlorophyll, proline, soluble sugar and malondialdehyde, root activity, field seedling emergence and the activities of superoxide dismutase, catalase, peroxidase and ascorbate peroxidase, the suitable biopriming treatments of SB-MGW9 for the three maize varieties were T2 (the seeds soaked with biostimulant for 3 h, and moisturized for 24 h) and T3 (the seeds soaked with biostimulant for 6 h, and moisturized for 12 h). SB-MGW9 could be a promising technique for decreasing the deleterious effects of salt stress for maize seed germination and seedling.

## Data Availability

The authors declare that all the data and materials used in this study are available.
